# Cardio-Respiratory Fitness and Autonomic Function in Patients with Major Depressive Disorder

**DOI:** 10.3389/fpsyt.2019.00980

**Published:** 2020-02-05

**Authors:** Marco Herbsleb, Andy Schumann, Luisa Lehmann, Holger H.W. Gabriel, Karl-Jürgen Bär

**Affiliations:** ^1^Department of Sports Medicine and Health Promotion, Friedrich-Schiller-University, Jena, Germany; ^2^Psychiatric Brain and Body Research Group, Department of Psychosomatic Medicine, University Hospital, Jena, Germany

**Keywords:** depression, autonomic function, exercise testing, cardio-respiratory fitness, vagal threshold, recovery, heart rate, heart rate variability

## Abstract

Patients with major depressive disorder (MDD) have an augmented risk of cardiovascular morbidity and mortality. Although a link between depression and autonomic dysfunction as well as reduced cardio-respiratory fitness (CRF) is well documented, the underlying cause is a matter of debate. Therefore, we studied the interplay between autonomic function, body composition and severity of the disease to disentangle possible physiological factors influencing the assumed lack of CRF in MDD patients. We investigated seventeen patients suffering from MDD and seventeen control subjects matched with respect to age, sex, body-mass-index, and smoking habits. A resting baseline assessment and a cardiopulmonary exercise test including a prolonged recovery period were performed to study autonomic function (i.e., heart rate responses and heart rate variability) during rest, exercise and recovery as well as CRF. Most investigated autonomic indices were significantly different at rest, during exercise as well as during recovery indicating altered autonomic modulation. Nevertheless, none of our participants was classified as chronotropically incompetent. As expected, a reduced CRF (i.e., peak oxygen uptake and peak power output, p < 0.01) was observed in patients compared to controls. In addition, a correlation of baseline heart rate and of heart rate during recovery with the ventilatory threshold 1 (p < 0.05) was found in patients only, indicating a relation to the lack of CRF. Furthermore, we observed a positive correlation of the severity of the disease with the weekly sitting time (p < 0.01) as well as a negative correlation with the activity time in the intensity domain walking (p < 0.001) and with the total score of the International Physical Activity Questionnaire (p < 0.01) for patients. This study shows that patients with MDD have altered autonomic function not only during resting conditions but also during exercise as well as recovery from exercise. Intervention studies are needed to evaluate how the described autonomic alterations can be influenced by increasing CRF due to appropriate exercise training programs.

## Introduction

Impaired mood, reduced energy, repetitive negative thinking and general loss of interest are key characteristics of major depressive disorder (MDD). According to the World Health Organization, depression is the fourth most common disease and one of the most relevant causes of disability worldwide (1, 2). The lifetime risk to suffer from depression was estimated between 15 and 19% (3).

MDD is closely linked to heart disease with significant clinical and economic consequences (4). Longitudinal cohort studies show that MDD subsequently increases the risk of cardiovascular morbidity and mortality (5, 6). MDD is already elevated as the first “psychological” variable to the status of an independent risk factor for a wide range of cardiovascular disease (CVD) in leading heart societies guidelines and recommendations (7–9). For instance, the risk of having coronary artery disease and myocardial infarction increases by a factor of about 1.6 for patients with MDD (10, 11). Interestingly, successful antidepressant treatment has not changed the increased mortality risk in depressed patients after myocardial infarction (12).

One important link between major depression and heart disease is the autonomic imbalance leading to sympathetic overstimulation and reduced parasympathetic function. Although many studies investigated the role of the autonomic nervous system (ANS) in depression, the definite involvement of both branches in the disease has not been described satisfactorily. The sympathetic nervous system is responsible for adjusting physical performance during stressful events or danger, whereas the parasympathetic nervous system is involved in regeneration, anabolism and conservation of resources. The heart is influenced by both peripheral branches of the ANS in a widely antagonistic fashion. Heart rate (HR) and its variability (HRV) are important markers of cardiac autonomic function. Nearly all measures of HRV are controlled by rapid vagal reflexes and reflect therefore parasympathetic modulation only.

Several studies reported low vagal function in unmedicated patients (13–15). Meta-analyses demonstrate a significant relation of depression and HRV decrease (16, 17). Antidepressant treatment has been reported to further decrease vagal modulation. In a longitudinal study, Licht et al. (18) showed that tricyclic, serotonergic as well as noradrenergic antidepressants are associated with a decrease in cardiac vagal function (18). In a recent study, we observed that autonomic differences of heart rate, skin conductance level and pupillary unrest index led to a 93% specificity to separate patients from controls highlighting the severity of autonomic dysfunction in patients with depression (19).

The role of exercise as a treatment modality for depression has been extensively studied. Exercise seems to reduce the severity of the major depressive episode and several meta-analyses have reported therapeutic effects of physical activity interventions in patients with clinical depression, with effects sizes ranging from 0.34 to 0.8 (20–23). Interestingly, the severity of depressive symptoms is inversely correlated with cardio-respiratory fitness (CRF) and this correlation is stronger in men than in women.

However, although reduced CRF is well documented in major depression (24–26) the underlying cause is a matter of debate. While moderators of effects of exercise interventions such as brain-derived neurotrophic factor (BDNF) or body mass index were suggested, the actual reason for reduced CRF in these patients is unknown and needs to be elucidated. One possible underlying cause might be related to autonomic dysfunction in the disease. Therefore, we studied the interplay between autonomic function, body composition and severity of the disease to disentangle possible physiological factors influencing the assumed lack of CRF in depressed patients.

## Materials and Methods

### Subjects

Seventeen patients suffering from MDD and seventeen healthy controls matched with respect to age, sex, body-mass-index, and smoking habits were included in the study (see **Table 1**). Patients were treated either at a specialized unit for affective disorders or in the outpatient Department of the University Hospital Jena, Germany. Diagnosis of MDD was established by a staff psychiatrist when symptoms of patients who were admitted to our inpatient wards fulfilled DSM-V criteria (Diagnostic and Statistical Manual of Mental Disorders, 5th edition, published by the American Psychiatric Association). The severity of depressive symptoms was assessed by the Hamilton Depression Rating Scale (HAMD-21) (27), rated by a psychiatrist as well as by the self-rating Beck Depression Inventory (BDI) (28). This study complied with the Declaration of Helsinki. All participants gave written informed consent to a protocol approved by the Ethics Committee of the University Hospital, Jena. Patients were informed that refusal to participate in this study would not affect future treatment.

**Table 1:** Epidemiological and clinical characteristics of participants.

	Controls Mean ± SD	Patients Mean ± SD	P value
**Epidemiological Data**			
Gender (female/male)	12/5	12/5	n.s.
Age (years)	40.3 ± 12.0	38.0 ± 11.7	n.s.
Body mass (kg)	71.8 ± 12.1	69.2 ± 9.0	n.s.
Height (cm)	175 ± 10	173 ± 9	n.s.
Body mass index (kg/m^2^)	22.1 ± 2.3	21.5 ± 3.2	n.s.
Body fat (%)	30.1 ± 8.6	28.4 ± 9.2	n.s.
Fat free mass	50.3 ± 13.0	49.4 ± 9.1	n.s.
**Education**			
8 years at school	0	0	n.s.
10 years at school	4	10	n.s.
12 years at school (A-Level)	13	7	n.s.
**Sport**			
No sport	4	7	n.s.
<2h/week	8	5	n.s.
2–5h/week	3	4	n.s.
>5 h/week	2	1	n.s.
**Smoking Behaviour**			
Smokers	5	6	n.s.
<5 Cigarettes/day	1	2	n.s.
5–10 Cigarettes/day	3	1	n.s.
>10 Cigarettes/day	1	3	n.s.
Fagerström test for nicotine dependence	0.6 ± 1.5	0.9 ± 1.8	n.s.
**International Physical Activity Questionnaire (IPAQ)**			
IPAQ-total (MET minutes/week)	2818 ± 1826	2288 ± 2074	n.s.
Vigorous physical activity (min/week)	1242 ± 1403	567 ± 1164	n.s.
Moderate physical activity (min/week)	875 ± 1271	619 ± 760	n.s.
Walking distance (METs min/week)	613 ± 737	1049 ± 891	n.s.
Time spent sitting (h/day)	6.6 ± 3.5	7.5 ± 4.3	n.s.
**Disease-related Data**			
Disease manifestation (years)	NA	4.21 ± 3.46	
Age of onset	NA	35.1 ± 11.8	
Current episode duration (weeks)	NA	6.0 ± 5.4	
BDI	2.9 ± 3.0	21.4 ± 8.1	<0.001
HAMD-21	NA	21 ± 7	
**Medication**			
SSRI	0	9	n.d.
SNRI	0	6	n.d.
NARI	0	1	n.d.

SD, standard deviation; NA, not applicable; n.d., not done; BDI, Beck's Depression Inventory; HAMD- 21, Hamilton Depression Rating Scale; AAP, atypical antipsychotics; SSRIs, selective serotonin reuptake inhibitors; SNRI, serotonin and norepinephrine reuptake inhibitors; NARI, noradrenaline reuptake inhibitors.

### Baseline Measurements

Prior to each test, standing height, body mass, and body fat were assessed using a harpenden stadiometer (Holtain Ltd, Crosswell, Crymych, UK), physician scale (SECA, Model 710, Hamburg, Germany), and harpenden skinfold caliper (Holtain Ltd, Crosswell, Crymych, UK), respectively. Skinfolds measurements were taken from the biceps, triceps, supra-illiac, and subscapular skinfold for male and females according to the published guidelines (29). Linear regression equations of Durnin & Wormersley (1974) (30) were used to predict body density. Percent of body fat was estimated using the equation of Siri (31). An electrocardiographic recording system (Schiller AT 10plus, Ottobrunn, Germany) that meets the specifications set by the American Heart Association (32) was applied before testing for continuous monitoring of heart rhythm and evaluation of ischemic electrocardiographic changes during exercise and recovery.

Subjects were then familiarized with the exercise testing equipment and procedures. First, a resting period of 5min in quite sitting on the electronically braked cycle-ergometer (ergoselect 100^®^, Ergoline, Bitz, Germany) was done before each test session to ensure a stable baseline. Blood pressure, body temperature, carbon monoxide in the exhaled breath (CO-Check^®^, Neomed GmbH, Korschenbroich, Germany) and blood lactate concentration were measured of all participants in the 4th minute at rest. Heart rate was continuously recorded beat-to-beat *via* a heart rate monitor (S610, Polar Electro Oy, Kempele, Finland) and averaged from the beginning of the second up to the end of the 4th minute (3-min interval) for data analysis.

### Exercise Testing Protocol

After the resting period, graded cardiopulmonary exercise test (CPET) was performed with 15 W/min increments until the subject reached his or her limit of tolerance preceded by a 3-min pedaling at 5 W. Exhaustion was deemed to have occurred when the participant could no longer maintain the required power output. We verbally encouraged subjects to aim for a pedaling-frequency of 70–80 revolutions per minute and to give maximum effort until volitional exhaustion.

Heart rate was continuously recorded throughout the test. Blood pressure were recorded at the last 30 sec of each 2-min interval and perceived exertion (6–20 Borg scale) at the last 5 sec of each minute throughout the exercise test. Because the degree of effort spent by the tested subject has an obvious influence on maximal performance indices, objective exhaustion levels were determined by measuring maximum lactate levels (La), using the respiratory exchange ratio (RER) of carbon dioxide (CO_2_) output to oxygen (O_2_) uptake, the highest reached HR, and the occurrence of a plateau in oxygen uptake. The degree of effort spent by the tested subject was further determined using the standardized subjective exhaustion 6–20 Borg scale (33). The maximal effort was deemed to be achieved if the incremental test met three of the following criteria: 1) a plateau in O_2_ uptake with increases in power output (O_2_ uptake-time slope <0,05 l/min for the final 30 sec), 2) The attainment of a RER_max_ ≥ 1.10, 3) a La_max_ ≥ 8 mmol · l^–1^, 4) the highest HR within ± 10 beats/min of age-predicted maximum HR (208-0,7 · age), and 5) a maximal Borg rating of perceived exertion ≥18. After a patient's individual limit of tolerance was reached, a cooling down phase ensued, consisting of 3-min pedaling at a slow rate (<40 revolutions/minute) at a power output of 15 W and the participant remained quite seated on the cycle-ergometer for another 7 min without pedaling.

### Physiological Regulation Parameters

#### Assessment of Cardio-Respiratory Fitness

During the resting period, the CPET, and recovery phase ventilatory indices and gas exchanges were measured continuously on a breath-by-breath basis using an automatic ergospirometer (MetaLyzer 3B, Cortex, Leipzig, Germany). Before each test, the turbine (flow and volume) was calibrated with a syringe (Hans Rudolph Inc, Kansas City). The gas analyzers were calibrated according to the manufacturer's guidelines with the same certified calibration gas mixture of 5% CO_2_ and 15% O_2_ (Air Liquide Healthcare America Corporation, Plumsteadville, PA). For data analysis, the breath-by-breath values were smoothed using a 15-breath moving average, aligned to the time of the central breath, which is recommended for improved data processing (34).

Baseline values of oxygen consumption ($\dot{V}O_{2}$), minute ventilation (${\dot{V}}_{E}$), respiratory frequency, and RER were stated as an average from the beginning of the second up to the end of the 4th minute of the resting period. Peak values of oxygen uptake ($\dot{V}O_{2peak}$), ventilation, respiratory frequency and RER were defined as the highest value of 15-breath average occurring during exercise test. In addition, we assessed the oxygen uptake at the ventilatory threshold 1 ($\dot{V}O_{2VT1}$, also termed aerobic gas exchange threshold), a submaximal indicator of endurance capacity. $\dot{V}O_{2VT1}$ was determined using a combined model according to Gaskill et al. including the following three methods: i) the V-slope method: the first disproportionate increase in $\dot{V}CO_{2}$ determined from the $\dot{V}CO_{2}/\dot{V}O_{2}$ plot, ii) the ventilatory equivalent method: an increase in ${\dot{V}}_{E}/\dot{V}O_{2}$ with no increase in ${\dot{V}}_{E}/\dot{V}CO_{2}$, and iii) the excess carbon dioxide method: an increase from steady state to an excess production of CO_2_ (35).

The peak power output (P_peak_) was defined as the highest power output that was sustained for 1 min during the test. When participants were not able to cycle to the end of the last 1-min interval, P_peak_ was linearly interpolated based on the proportion of the time completed during the terminal stage.

#### Blood Lactate

Capillary blood samples of 20 μl were obtained from the ear lobe when the participant was at rest, and again 1 and 3 min after the end of the exercise test. The lactate concentration in millimoles per liter was measured by the EBIO basic system analyzer, using an enzymatic-amperometric measuring system (Eppendorf, Hamburg, Germany).

#### Heart Rate Variability (HRV)

Heart rate time series consisting of successive beat-to-beat intervals (RR) were extracted from the raw data records. Afterwards, these time series were filtered by applying an adaptive variance estimation algorithm to remove and interpolate ventricular premature beats and artifacts (e.g., movement, electrode noise, and extraordinary peaks). The RR from the second up to the 4th minute of rest as far as from the eighth up to the 10th minute of the recovery period were used for analysis. We obtained the baseline HR and the root mean of squared successive difference (RMSSD) as a time domain parameter of HRV. Nonlinear properties of heart rate were investigated using the poincaré plot. A return map is constructed by plotting each RR against the preceding RR. The variance of the resulting scatter gram is characterized by the deviations SD1 and SD2. Here we focused on the instantaneous variability reflected by SD1, which is related to parasympathetic activity (36). Because of a strong dependency of SD1 on basic heart rhythm we also estimated this index normalized by mean RR (SD1/RR_Mean_) (37).

#### Determination of Vagal Threshold

HRV time series were calculated using non-overlapping windows of 60 sec duration. In order to optimize temporal resolution, we additionally applied an event-based approach defining a sliding window of 30 RR shifted by one RR. For calculation of the vagal threshold (VT), values of the instantaneous variability in RR (SD1) from the Poincaré plot (see above) were plotted in relation to power output using Matlab 2011 (The Math Works Inc, Natick) as described previously by (38). The VT is the point in time at which no subsequent decline in HRV occurs, indicating the moment when parasympathetic activity has decreased and sympathetic activation will increase, in such a way that almost no vagal modulation remains. When the VT is passed, further increase of power output does not lead to a further decline of parasympathetic activity. Mean value plus 3 times the standard deviation of the last 30% of the recording was used to define the deflection point of the HRV time course. When SD1 drops below this value VT is reached.

#### Assessment of Heart Rate Responses to Exercise

To assess chronotropic response, the metabolic-chronotropic relationship (MCR; also known as the chronotropic index) was calculated according to Wilkoff et al. (39) using the ratio of heart rate reserve [predicted HR_peak_ (220- age)—HR at rest] to metabolic reserve ($\dot{V}O_{2peak}-\dot{V}O_{2}$ at rest) during the CPET. The MCR adjusts for age, physical fitness and functional capacity and is unaffected by the exercise protocol as well as effort-independent (40). The increase in percentage heart rate reserve in relation to the increase in percentage metabolic reserve (also known as oxygen uptake reserve) was obtained by linear regression analysis using the least squares method of the mean values acquired for $\dot{V}O_{2}$ and HR in the last 30 s of each incremental step of the CPET. In healthy adults, the percentage of heart rate reserve achieved during exercise equals the percentage of metabolic reserve achieved, so that the median MCR slope is near around 1.0 with a 95% confidence interval between 0.8 and 1.3 (39).

### Statistics

For statistical analysis, SPSS for Mac (version 25.0.0.1) was used. All parameters were tested for normal distribution with the Shapiro-Wilk test.

Because a strong statistical relationship between increases in fat free body mass (FFM) and indices of maximal exercise performance ($\dot{V}O_{2\text{peak}}{\text{ and P}}_{\text{peak}}$) exists and several studies have shown that FFM is the best single predictor of $\dot{V}O_{2\text{peak}}$ (41, 42), we performed a multivariate analysis of covariance (MANCOVA) with the between-subject factor GROUP (patients and controls) and FFM as a covariate for $\dot{V}O_{2\text{peak}}$, $\dot{V}O_{2VT1}$ and P_peak_, followed by univariate ANOVAs for both parameters. One assumption for using least squares linear modeling techniques is that the slopes of the regression lines (e.g. $\dot{V}O_{2\text{peak}}$ vs. FFM) for both groups must be constrained to be parallel. Therefore, the slope (b) term for both groups would be statistically compared using the standard statistical technique analysis of covariance (ANCOVA), a combination of regression and analysis of variance. The statistical comparison of the intercept terms reflects any difference in the magnitude of peak oxygen uptake resp. peak power output between the groups.

Further, a repeated measures MANOVA was performed to investigate an overall effect for the factors GROUP (patients and controls), TIME (baseline and recovery) and GROUP × TIME interaction for the physiological parameters (HR, RMSSD, SD1, and SD1/RR_Mean_).

To investigate the metabolic–chronotropic relationship slope (MCR slope) of patients and controls was analyzed. Controls and patients with an MCR slope below the cut-off value of 0.8 were referred as chronotropic incompetent whereas subjects with an MCR slope above a value of 0.8 were classified as chronotropic competent (39). Group differences of the MCR slope between patients and controls were verified with a parametric two-sample t-test.

To account for possible differences between groups in vagal threshold and heart rate recovery we performed a MANOVA for threshold (VT-SD1 and VT-SD1/RR_Mean_) and recovery data (HRR_1min_ and HRR_8-10min_). All HRV-parameters were log-transformed using natural logarithms before being included in analysis to achieve normal distribution after the Shapiro-Wilk test had indicated non-normal distribution.

To relate aerobic capacity to observed autonomic measures, we correlated $\dot{V}O_{2\text{peak}}$ and $\dot{V}O_{2\text{VT}1}$ (both normalized to FFM), to heart rate and heart rate variability (HR and SD1/RR_Mean_) at rest and during recovery phase, as well as to the MCR slope for both groups separately using the Pearson correlation coefficient.

Finally, Spearman's rank correlation coefficient was calculated to investigate a potential correlation of the amount and intensity of physical activity (IPAQ-domains) of patients with the severity of depression as assessed by BDI and HAMD-21 scores.

## Results

### Physical Fitness

**Figure 1:**
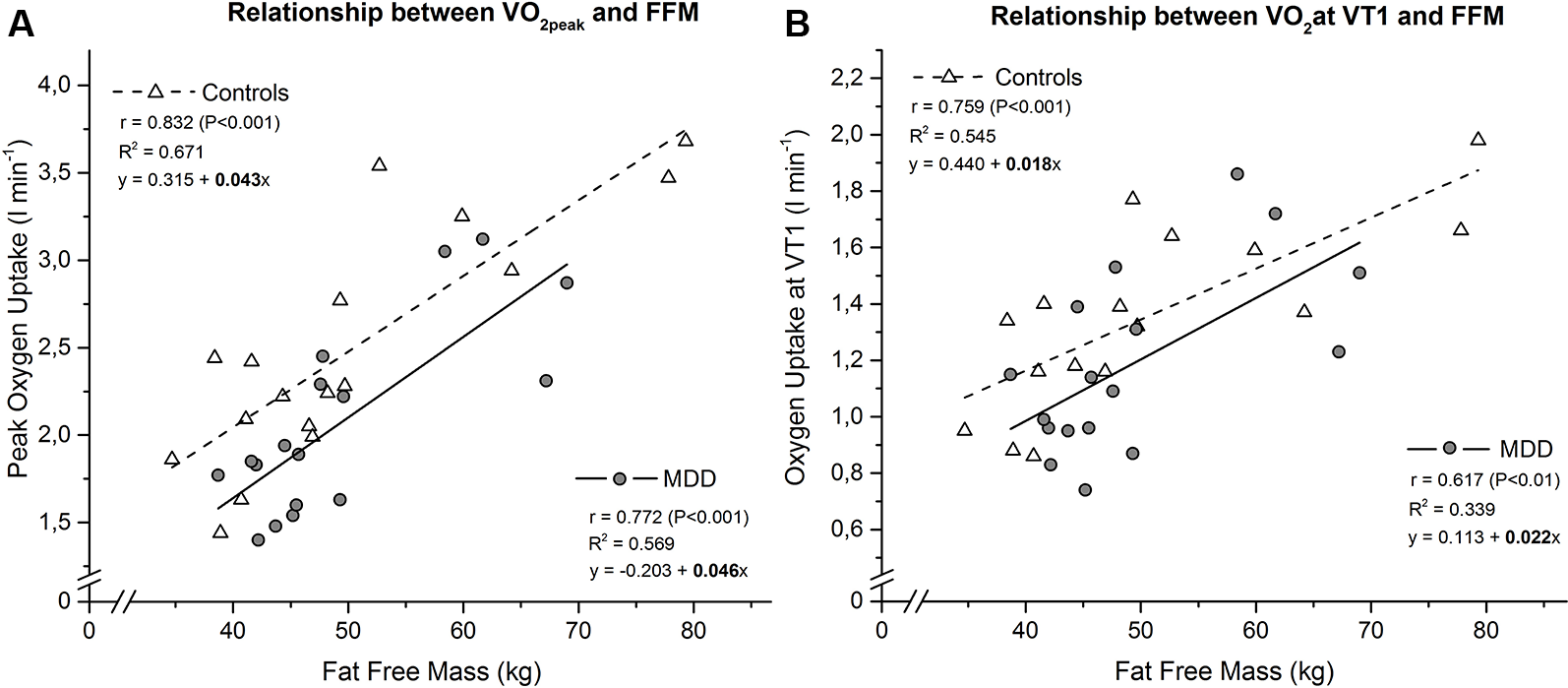
Linear relationship between peak oxygen uptake **(A)** resp. oxygen uptake at ventilatory threshold 1 **(B)** and fat free mass in MDD versus controls. The slope coefficients for controls (b = 0.043 for $\dot{V}O_{2\text{peak}}$ and b = 0.018 for $\dot{V}O_{2\text{VT}1}$) and MDD (b = 0.046 for $\dot{V}O_{2\text{peak}}$ and b = 0.022 for $\dot{V}O_{2\text{VT}1}$) were not significant different between groups (p = 0.832 for $\dot{V}O_{2\text{peak}}$ and p = 0.642 for $\dot{V}O_{2\text{VT}1}$). Subsequent comparison of the intercept terms for controls (a = 0.315 for $\dot{V}O_{2\text{peak}}$ and a = 0.440 for $\dot{V}O_{2\text{VT}1}$) and MDD (a = -0.203 for $\dot{V}O_{2\text{peak}}$ and a = 0.113 for $\dot{V}O_{2\text{VT}1}$) revealed a significant difference, confirming the higher peak oxygen uptake (P < 0.01) and a trend for higher oxygen uptake at ventilatory threshold 1 (P < 0.1) of the controls.

All tested fitness-parameters showed normal distribution according to the Shaprio-Wilk test. The performed MANCOVA revealed a significant effect for the between-subject factor GROUP (F(3,28) = 3.634, p = 0.025). Follow-up univariate ANOVAs showed significant differences for $\dot{V}O_{2\text{peak}}$ (p < 0.01) and P_peak_ (p < 0.01), as well as a trend for $\dot{V}O_{2\text{VT}1}$ (p < 0.1) indicating lower fitness levels in patients (**Figure 1**, **Table 2**).

**Table 2:** Peak values and oxygen uptake at ventilatory threshold 1 attained during ramp exercise test.

	Controls Mean ± SD	Patients Mean ± SD	P value
Power output (W)	204 ± 46	171 ± 43	<0.05
Power output (W) – adjusted for FFM*	202 (7.3)	172 (7.3)	<0.01
Oxygen uptake (L min^-1^)	2.49 ± 0.68	2.07 ± 0.54	n.s.
Oxygen uptake (L min^-1^) – adjusted for FFM*	2.47 (0.089)	2.09 (0.089)	<0.01
Minute ventilation (L min^-1^)	104.5 ± 23.5	85.1 ± 20.3	<0.05
Breathing rate (breaths min^-1^)	46 ± 10	40 ± 8	<0.05
Heart rate (beats min^-1^)	182 ± 11	177 ± 15	n.s.
Lactate concentration (mmol L^-1^)	9.6 ± 1.6	9.1 ± 2.4	n.s.
Respiratory exchange ratio	1.23 ± 0.11	1.25 ± 0.11	n.s.
Rating of perceived exertion (BORG 6-20 scale)	19.4 ± 0.9	19.1 ± 0.9	n.s.
Oxygen uptake at ventilatory threshold 1 (L min^-1^)	1.35 ± 0.32	1.19 ± 0.32	n.s.
Oxygen uptake at ventilatory threshold 1 (L min^-1^)—adjusted for FFM*	1.34 (0.058)	1.20 (0.059)	<0.1

*Peak power output, peak oxygen uptake, and oxygen uptake at ventilatory threshold 1 has been adjusted for the influence of fat free mass.

Data are presented as the mean ± SD and as mean (SE) for adjusted values.

### Metabolic–Chronotropic Relationship

**Figure 2:**
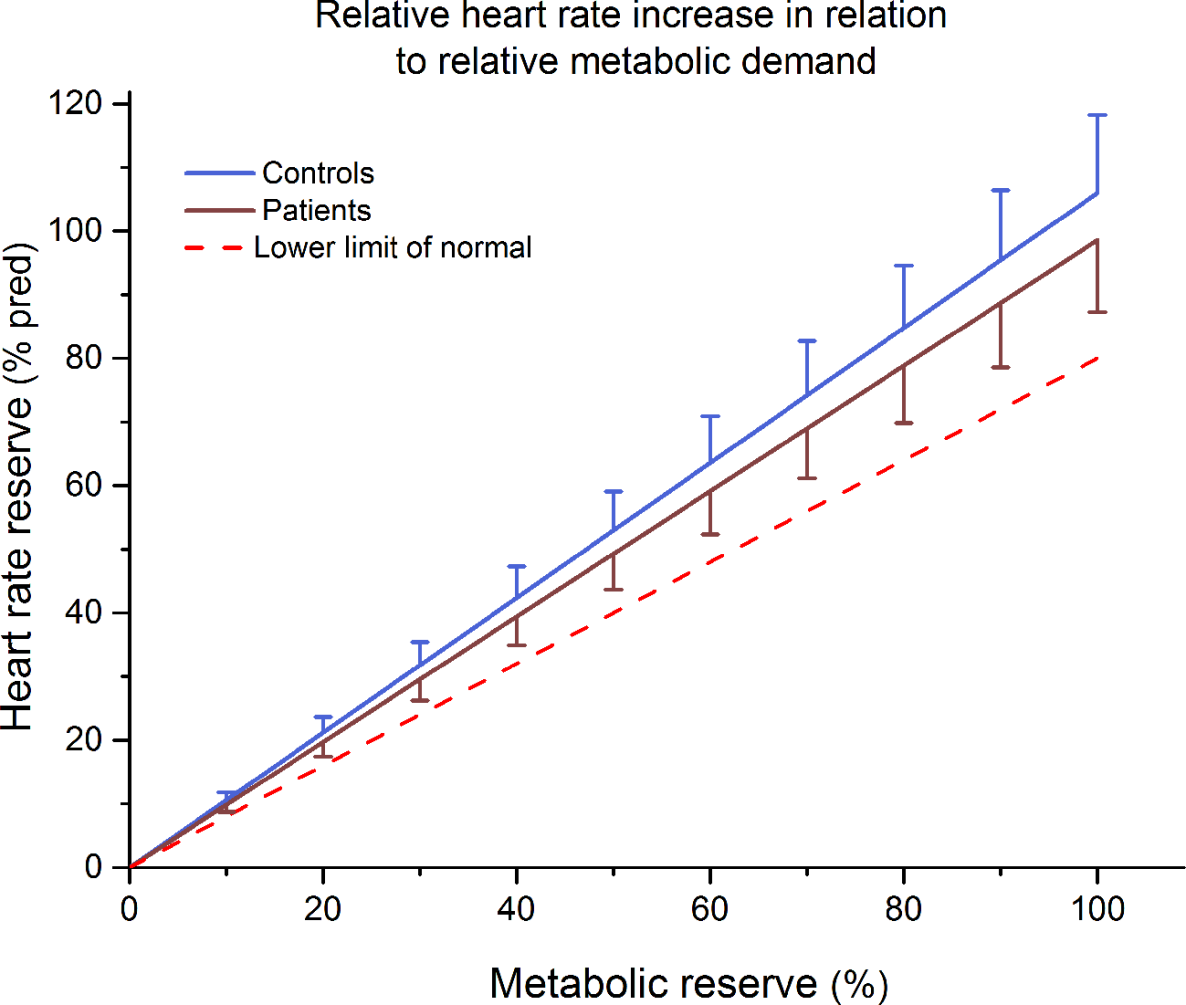
Mean ± 90% confidence limit of the increase in percentage heart rate reserve in relation to the increase in percentage metabolic reserve of patients and control subjects. The dotted line represents the lower limit of normal (slope b = 0.8).

None of our participants was classified as chronotropically incompetent according to cut-off values (39, 40). Furthermore, no significant differences were observed between mean MCR slopes of patients and controls (0.99 ± 0.13 and 1.06 ± 0.14 respectively; p > 0.05) (**Figure 2**).

### HRV

**Figure 3:**
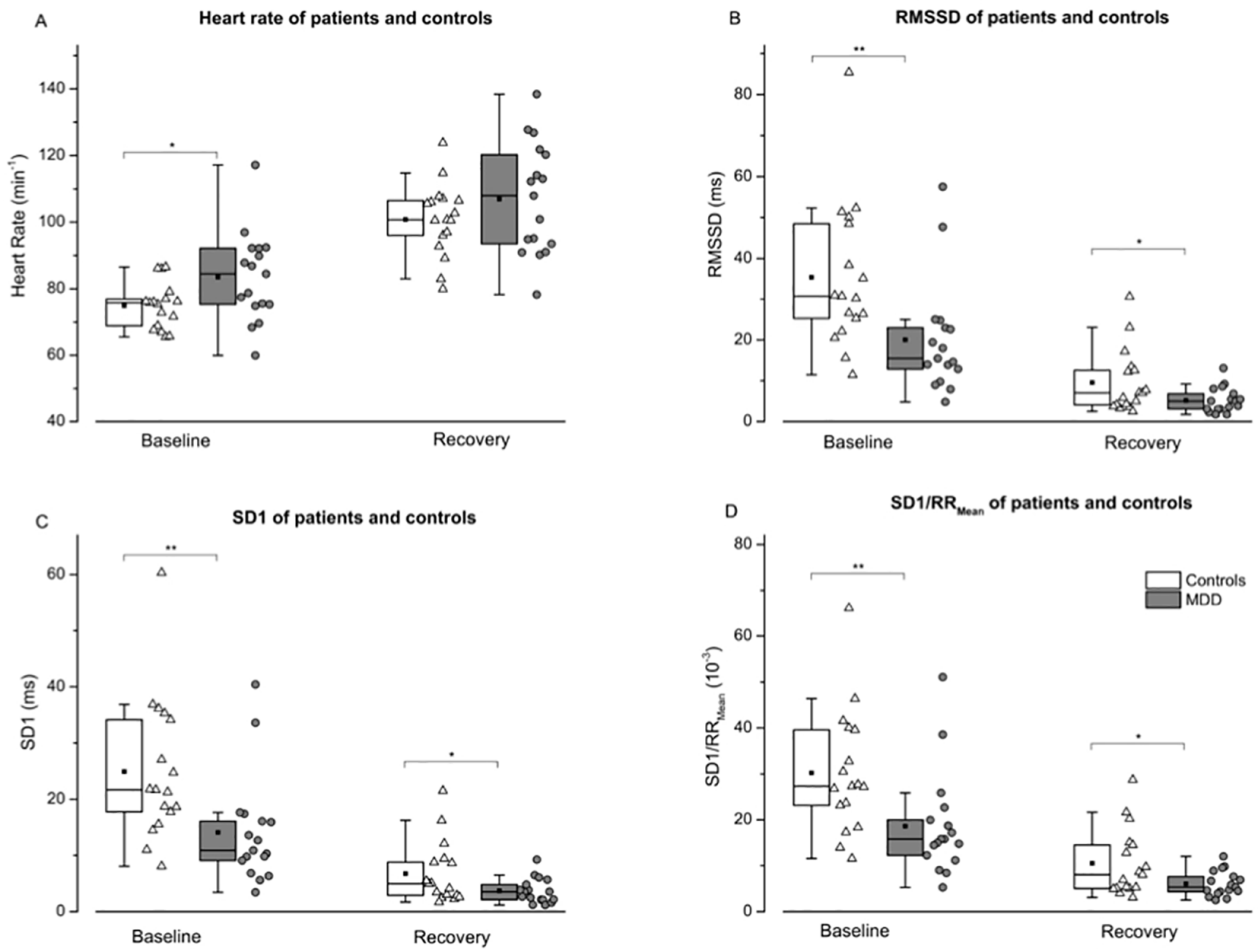
Heart rate **(A)**, root mean of squared successive difference—RMSSD **(B)**, instantaneous variability—SD1 **(C)**, and SD1 normalized by mean RR—SD1/RR_Mean_ **(D)** are shown for controls (white boxes resp. triangles) and patients (grey boxes resp. points) before and 10 min after a maximal incremental exercise task. An independent two-tailed t test was calculated to compare patients and controls for descriptive analysis. While differences between heart rate were only shown at rest (A), reduced variability (B, C and D) were evident before and after exercise. Boxes indicate data between the 25th and 75th percentile with the horizontal bar reflecting the median (▪ = mean; – = 1.5 x interquartile range). Significant differences of pair-wise comparisons are indicated: *P < 0.05; **P < 0.01.

The repeated measures MANOVA for HR and HRV values (RMSSD, SD1 and SD1/RR_Mean_) indicated an overall effect for the factor GROUP [F(4,29) = 4.611, P < 0.01], a significant effect for the factor TIME [F(4,29) = 45.662, P < 0.001] but no significant effect for the TIME × GROUP interaction [F(4,29) = 1,467 p = 0.238]. This is suggestive for differences of physiological parameters between both groups. For specific parameters *post hoc* ANOVAs revealed a significant effect for the factor GROUP for RMSSD (F = 11.262, p < 0.005), SD1 (F = 11.203, p < 0.005), SD1/RR_Mean_ (F = 11.812, p < 0.005), and a non-significant trend for HR (F = 3.736, p = 0.062). In addition, we observed significant differences for the factor TIME (baseline vs. recovery) for HR (F = 160.0, p < 0.001), RMSSD (F = 121.4, p < 0.001), SD1 (F = 121.4, p < 0.001), and for SD1/RR_Mean_ (F = 97.102, p < 0.001). The single points in time (baseline and recovery) are depicted in **Figure 3**.

**Figure 4:**
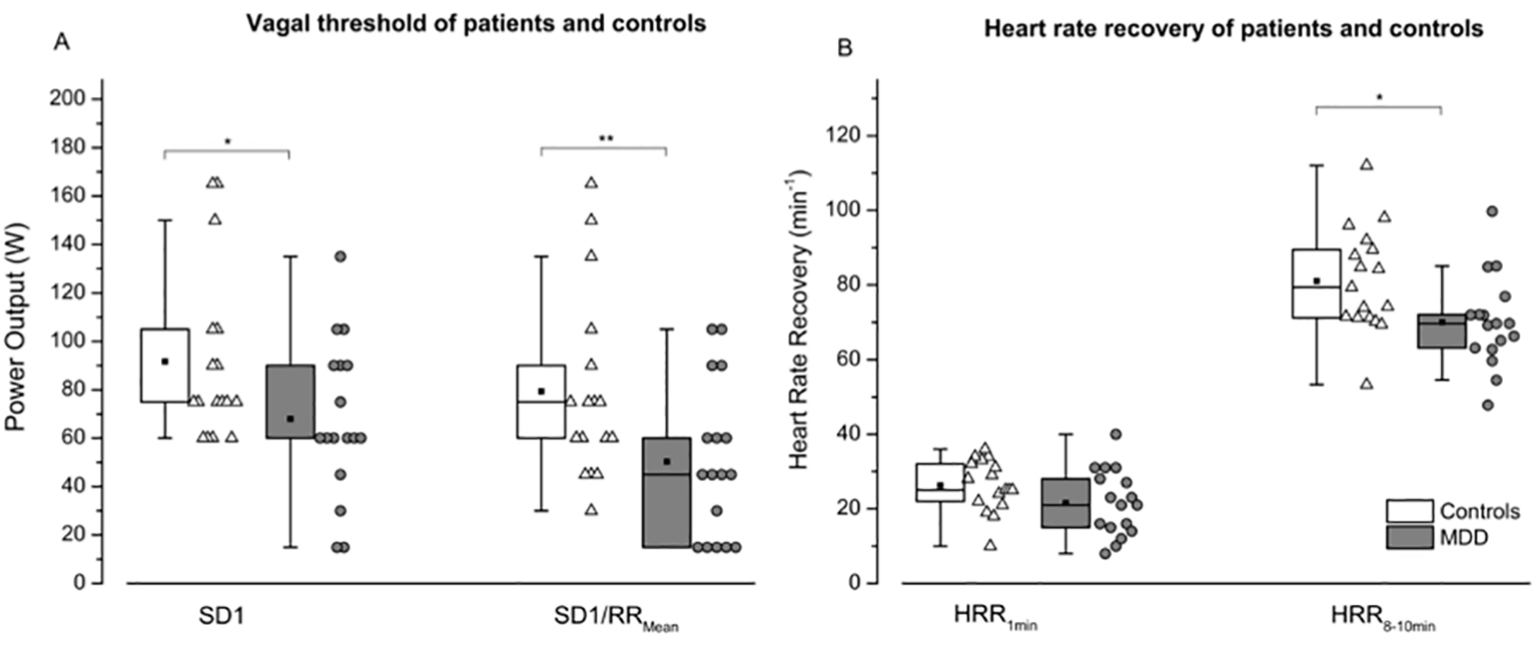
Vagal threshold **(A)** and heart rate recovery **(B)** are shown for controls (white boxes resp. triangles) and patients (grey boxes resp. points) following maximal exercise. SD1: instantaneous variability; SD1/RR_Mean:_ SD1normalized by mean RRI; HRR_1min_: Heart rate recovery 1 min after exercise secession; HRR_8-10min_: Heart rate recovery 8–10 minutes after exercise secession. Boxes indicate data between the 25th and 75th percentile with the horizontal bar reflecting the median (▪ = mean; – = 1.5 x interquartile range). Significant differences of pair-wise comparisons are indicated: *P < 0.05; **P < 0.01.

The MANOVA for vagal threshold (VT-SD1 and VT-SD1/RR_Mean_) and heart rate recovery (HRR_1min_ and HRR_8-10min_) data revealed a significant effect [F (4, 29) = 2.055; p < 0.05] for the factor GROUP (patients vs. controls). The follow-up univariate ANOVAs indicated significant differences for VT-SD1 (F = 5.051; p < 0.05), VT-SD1/RR_Mean_ (F = 7.663; p < 0.01) as well as for HRR_8-10min_ (F = 6.068; p < 0.05), and a non-significant trend for HRR_1min_ (F = 2.906; p = 0.098; **Figure 4**).

### Correlation Between Autonomic Function and CRF

We observed a negative correlation of baseline heart rate with $\dot{V}O_{2VT1}$ (r = -0.521, p < 0.05) in patients. No such relation was found in healthy subjects. In addition, we observed for patients a significant positive correlation between HRR_8-10min_ and $\dot{V}O_{2VT1}$ (r = 0.516, p < 0.05). We did not detect any further correlation between autonomic function and CRF in patients and not any in the control group.

### Correlation Between Physical Activity/Inactivity and Severity of Disease

Furthermore, we observed a positive correlation of the BDI with the IPAQ reported sedentary activity (sitting time) for the week (r = 0.693, p < 0.01) and a negative correlation between the Hamilton rating scale and the IPAQ reported activity time in the intensity domain walking (r = -0.804, p < 0.001) as well as the total IPAQ score (r = -0.742, p < 0.01) for patients.

## Discussion

Reduced CRF has been reported in patients suffering from major depressive disorder. The previously described correlation between symptom severity of patients and CRF suggests the existence of a physiological link (43). The question needs to be answered what might be the underlying relation between reduced CRF and the depressive state. Here, we report that indeed patients suffering from MDD show reduced physical fitness. However, there was no relation to body fat or any other obvious reason. Since previous research suggested that depressed individuals tend to be less active than non-depressed individuals and this might account for the reduced fitness, we applied the International Physical Activity Questionnaire (IPAQ). As shown in **Table 1**, there was no difference in mean values of the total score, vigorous exercises, walking distance or the sitting time. This makes a general statement of differences in daily activity less likely. However, we observed interesting relations between IPAQ and the severity of depression as assessed by the BDI or Hamilton depression rating scale. Thus, more severely depressed patients were less physical active. However, no relation to the level of CRF could be demonstrated.

In addition, it has been assumed that depressed individuals may actually feel that they have reached fatigue during stress testing sooner than normal because of somatic symptoms of depression. Here we show that participants did not differ with respect to the degree of effort during the incremental exercise test (**Table 2**, and **Table 1** in the **Supplementary Material**). We used the objective exhaustion criteria peak heart rate, peak lactate levels, the peak respiratory exchange ratio, levelling in O_2_ uptake, as well as the assessment of the subjective perceived exertion using the Borg scale. Furthermore, we assessed the $\dot{V}O_{2VT1}$, an effort and motivation independent submaximal indicator of CRF. Thus, we are inclined to exclude the possibility that reduced CRF is caused by different fatigue levels during the CPET test.

Reduced CRF is in some patient populations associated with impaired heart rate response to physical exercise. We assessed therefore a possible link between autonomic function and CRF. As described in the introduction, there are many studies pointing towards disturbed autonomic regulatory mechanisms in the disease. Both branches, the vagal and sympathetic system have been shown to be involved in cardio-vascular dysregulation in the disease. As shown in **Figure 3**, we observed a significant degree of autonomic dysfunction in patients at baseline. Here, increased heart rates were observed accompanied by reduced vagal modulation (RMSSD or SD1). Even the adjustment of SD1 to mean heart rates did not alter the result. Thus, autonomic dysfunction is present in our patients at rest and might be influential on CRF. However, peak heart rates were similar in both groups, excluding the presence of chronotropic incompetence (CI) as described in other disease such as patients with schizophrenia (44, 45). CI is defined as the inability of the heart to increase its beating frequency in proportion to increased physical activity or higher metabolic demand. It is an established independent cardiovascular risk factor for major cardiac events and overall mortality and might explain adaptation intolerance of the cardiovascular system to even minor exercise courses. As shown in **Figure 2**, no such inability was observed in our patients. However, significant differences of vagal modulation were also present during recovery and are demonstrated in **Figures 3** and **4**.

Following the end of exercise, there is a progressive reduction in metabolic demand and, as a consequence, heart rate decreases. Heart rate recovers in a nearly first-order exponential fashion, with a fast decay in heart rate immediately after exercise (i.e., fast phase), followed by a more gradual decay (i.e., slow phase) until heart rate reaches its baseline values (46). Studies using pharmacological blockade have consistently shown that the fast phase of heart rate recovery is determined by parasympathetic reactivation, despite maintained sympathetic activation, whereas the later slow phase is predominately determined by additionally sympathetic withdrawal (47–49). Post-exercise HRR is therefore a simple non-invasive measurement related to autonomic nervous system dysfunction that indicates impaired parasympathetic reactivation and/or sympathetic withdrawal after exercise (46, 47, 50, 51). Previous studies have shown that blunted HRR_1min_, defined as a ≤ 12 beats/min decrease in heart rate from peak exercise to 1 min into recovery, is a powerful predictor of cardiovascular morbidity and overall mortality, even in asymptotic subjects (52). Furthermore, HRR_1min_ has been reported to be an independent predictor of endothelial function, and delayed due to autonomic dysfunction or imbalance (53). However, in our patient population we only observed a non-significant trend for lower HRR_1min_. The significant lower HRR_8-10min_ observed in our patients, could be in part a consequence of the higher baseline heart rate in this group due to sympathetic modulation. Interestingly, we found a correlation of baseline HR as well as HRR_8-10min_ with $\dot{V}O_{2VT1}$. Schumann and colleagues (19) suggested that the sympathetic overactivity might be the key alteration of the aberrant autonomic modulation in MDD. Our results indicate that sympathetic predominance might be related to reduced CRF in these patients. However, in contrast to patients with schizophrenia (44, 45) we have not observed any further correlation between other indices of autonomic function (e.g. HRV or CI) and CRF, suggesting that the impact of the autonomic state on CRF might be less severe.

Some limitations need to be addressed. First of all, the relatively small sample size of patients needs to be acknowledged. A second limitation pertains to the baseline measurement of heart beat derived indices. The baseline measurement take place during a 5-min period of sitting on the cycle-ergometer when patients were wearing a face mask for respiratory gas exchange measurement which might have influenced heart rate and its variability. So, some caution should be taken to transfer our results to real resting state measurements. At third limitation concerns the exercise test protocol and followed recovery period. All participants performed the same ramp-like exercise test protocol (starting from 15 W with 1min steps and increments of 15 W) which results in different exercise durations depending on participant's aerobic capacity (controls 816 ± 184 s, patients 684 ± 172 s). As the recovery of heart rate is mainly depending on exercise intensity but also to some extend on exercise duration, it might be that subjects who performed a longer test have potentially a slower HRR. However, controls exercised longer and the difference in exercise time was small. More importantly, the maximal effort was similar between groups and it is unlikely that test duration had such a meaningful influence on our recovery results. Finally, it has to be considered, that patients were treated with serotonergic or noradrenergic reuptake inhibitors (SSRI, SNRI, or NARI). There are some studies suggesting a significant effect of antidepressants on vagal cardiac regulation (15, 54). However, a meta-analysis showed that these types of antidepressants have minimal impact on vagal cardiac function (17).

In conclusion, this study shows that the shift of autonomic balance towards sympathetic predominance in patients with MDD in not only present during resting state conditions but also in some extend during physical exercise as well as during recovery from physical high demanding tasks. Furthermore, a correlation of ventilatory threshold 1 with baseline heart rate and the heart rate at the slow phase of heart rate recovery suggests a link between reduced CRF and autonomic function. Intervention studies are needed, to further evaluate if described autonomic alterations can be influenced by increasing the level of CRF due to appropriate exercise training programs.

## Data Availability Statement

The datasets generated for this study are available on request to the corresponding author.

## Ethics Statement

The studies involving human participants were reviewed and approved by the Ethics Committee of the University Hospital, Jena, Germany. The patients/participants provided their written informed consent to participate in this study.

## Author Contributions

MH: performed testing procedure, data calculation and writing the manuscript. AS: autonomic analysis. LL: exercise testing and calculation of data. HG and K-JB: leading the study, writing manuscript and data interpretation.

## Conflict of Interest

The authors declare that the research was conducted in the absence of any commercial or financial relationships that could be construed as a potential conflict of interest.
