# Supplementary material for: Cardio-Respiratory Fitness and Autonomic Function in Patients with Major Depressive Disorder
Source: Front Psychiatry. 2020 Feb 5;10:980. doi: 10.3389/fpsyt.2019.00980 (PMC7011194; doi:10.3389/fpsyt.2019.00980)
Supplement: Supplementary file 1 [file Table_1.docx]

Supplementary Material

# Supplementary Table

Criteria for evaluating the degree of effort on the incremental exercise test. Number of patients and controls that met the accepted criteria for maximal effort are shown.

|  | Controls  yes/no | Patients  yes/no | P value |
| --- | --- | --- | --- |
| Predicted heart rate ± 10 beats | 15/2 | 13/4 | n.s. |
| Lactate concentration ≥ 8mmol/L | 15/2 | 12/5 | n.s. |
| Respiratory exchange ratio ≥ 1.10 | 15/2 | 16/1 | n.s. |
| Plateau in oxygen uptake | 10/7 | 8/9 | n.s. |
| Rating of perceived exertion scale ≥ 18 | 16/1 | 16/1 | n.s. |
| **Met three of the five exhaustion criteria** | **16/1** | **14/3** | **n.s.** |

The dichotomy-type data were analysed using Fisher's exact test.
